# Identifying the appropriate time for deep brain stimulation to achieve spatial memory improvement on the Morris water maze

**DOI:** 10.1186/s12868-017-0345-4

**Published:** 2017-03-07

**Authors:** Da Un Jeong, Jihyeon Lee, Won Seok Chang, Jin Woo Chang

**Affiliations:** 1 Brain Korea 21 PLUS Project for Medical Science and Brain Research Institute, Yonsei University College of Medicine, Seoul, Korea; 2 Department of Neurosurgery, Yonsei University College of Medicine, CPO Box 8044, Seoul, Korea

**Keywords:** Deep brain stimulation, Spatial memory, Brain-derived neurotrophic factor

## Abstract

**Background:**

The possibility of using deep brain stimulation (DBS) for memory enhancement has recently been reported, but the precise underlying mechanisms of its effects remain unknown. Our previous study suggested that spatial memory improvement by medial septum (MS)-DBS may be associated with cholinergic regulation and neurogenesis. However, the affected stage of memory could not be distinguished because the stimulation was delivered during the execution of all memory processes. Therefore, this study was performed to determine the stage of memory affected by MS-DBS. Rats were administered 192 IgG-saporin to lesion cholinergic neurons. Stimulation was delivered at different times in different groups of rats: 5 days before the Morris water maze test (pre-stimulation), 5 days during the training phase of the Morris water maze test (training-stimulation), and 2 h before the Morris water maze probe test (probe-stimulation). A fourth group of rats was lesioned but received no stimulation. These four groups were compared with a normal (control) group.

**Results:**

The most effective memory restoration occurred in the pre-stimulation group. Moreover, the pre-stimulation group exhibited better recall of the platform position than the other stimulation groups. An increase in the level of brain derived neurotrophic factor (BDNF) was observed in the pre-stimulation group; this increase was maintained for 1 week. However, acetylcholinesterase activity in the pre-stimulation group was not significantly different from the lesion group.

**Conclusion:**

Memory impairment due to cholinergic denervation can be improved by DBS. The improvement is significantly correlated with the up-regulation of BDNF expression and neurogenesis. Based on the results of this study, the use of MS-DBS during the early stage of disease may restore spatial memory impairment.

## Background

Several therapies have been investigated in response to the growing prevalence of dementia. Several studies have reported that deep brain stimulation (DBS) of memory-associated brain structures is a promising potential treatment for dementia. Hypothalamic/fornix-DBS enhances some memory functions and modulates limbic activity [1, 2]. Entorhinal DBS during learning improves spatial memory [3]. Nucleus-basalis-of-Meynert-DBS also enhances cognitive function in patients with Parkinson patients [4]. However, the mechanism by which DBS enhances memory remains unclear. Therefore, animal studies that investigate these mechanisms are necessary.

Degeneration of cholinergic basal forebrain neurons, including those in the medial septum (MS), is a common feature of Alzheimer’s disease (AD) and vascular dementia, and has been correlated with cognitive decline [5, 6]. The MS projects its neuronal fibers, which include cholinergic, gamma-aminobutyric acid-ergic (GABAergic), and glutamatergic fibers, to the hippocampus [7, 8], and modulates hippocampal activity via acetylcholine, GABA, and glutamate release [9, 10]. Therefore, the current study was performed in a memory-impaired rat model with cholinergic denervation. In our previous study, we showed that 2 weeks of MS-DBS improved spatial memory in a memory-impaired rat model [11]. The results of this previous experiment suggest that spatial memory improvement by MS-DBS may be associated with cholinergic regulation and neurogenesis. However, the affected stage of memory (i.e., acquisition, consolidation, or retrieval) could not be distinguished because the stimulation was delivered while all memory processes were undergoing. In this study, to detect the stage of the memory process affected by MS-DBS, stimulation was delivered at different time intervals: 5 days before the Morris water maze test (pre-stimulation), 5 days during the training phase of the Morris water maze test (training-stimulation), 2 h before the Morris water maze probe test (probe-stimulation). Determination of the stage of memory affected by DBS can help identify the most effective time of stimulation for memory enhancement therapy.

## Methods

### Animals

This study was performed in accordance with the guidelines for the care and use of laboratory animals of the Institutional Animal Care and Use Committee of Yonsei University (IACUC number: 2014-0206). Rats were housed in a temperature- and humidity-controlled room with a 12:12 h light/dark cycle, and all rats had free access to food and water.

Eight weeks old forty-one male Sprague-Dawley rats (200–250 g) were randomly assigned to one of the five groups. Rats in the normal group (n = 8) underwent no surgical procedures. Rats in the lesion group (n = 8) and all stimulation groups received intraventricular administration of 192 IgG-saporin. In addition, rats in all the stimulation groups had an electrode implanted in their MS. Rats in the pre-stimulation group (n = 9) received stimulation for 5 days prior to the Morris water maze training. Rats in the training-stimulation group (n = 9) received stimulation for 5 days during the training phase of the Morris water maze test. Rats in the probe-stimulation group (n = 7) received stimulation for 2 h before the Morris water maze probe test.

### Surgical procedure and stimulation parameters

**Fig. 1:**
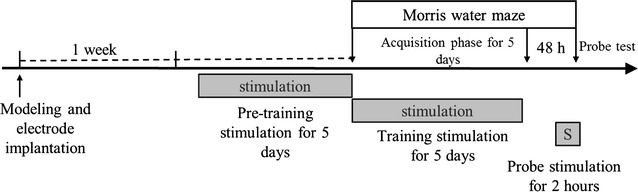
Schematic diagram of the stimulation and behavioral test timing. The pre-stimulation group received stimulation for 5 days prior to the water maze training. The Morris training-stimulation group received stimulation for 5 days during the Morris water maze training phase. The probe-stimulation group received stimulation for 2 h shortly before the Morris water maze probe test

Thirty-three rats were anesthetized with a mixture of ketamine (75 mg/kg), acepromazine (0.75 mg/kg), and rompun (4 mg/kg) and secured in a stereotaxic frame. After a scalp incision, rats were injected bilaterally with 8 µl of 192 IgG-saporin (0.63 µg/µl, Chemicon, Temecula, CA, USA) at the cerebroventricle based on the following coordinates from the bregma: anterior posterior (AP): −0.8 mm, medial lateral (ML): ±1.2 mm, dorsal ventral (DV): −3.4 mm. The solution was delivered at a rate of 1 µl/min using a syringe pump (Legato 130, KD Scientific, Holliston, MA, USA). The syringe was left in place for 5 min after the injection. After the administration of 192 IgG-saporin, 25 rats (all stimulation groups) underwent an additional procedure for electrode implantation. A hole was drilled in the skull at the level of the MS (AP: +0.6 mm, ML: 0.1 mm, DV: −6 mm from the bregma), and a unipolar tungsten electrode (254 µm diameter, A-M systems, Sequim, WA, USA) was implanted in the MS. The stimulation electrode was fixed with dental cement (Lang Dental Manufacturing, Wheeling, IL, USA). Following surgery, wounds were treated daily with Betadine. If a rat had an infection following surgery, cefazolin (4 mg/100 g) was administered intravenously for 3 days. The electrode was connected to a stimulator (Pulsemaster A300, stimulus isolator A365, WPI, Worcester, MA, USA). Electrical stimulation consisted of pulses (120 µs, 100 µA) delivered at 60 Hz. Stimulation was delivered as shown in the schematic diagram in Fig. 1. The Pre-stimulation group was stimulated for 5 consecutive days before the training phase (2 h/day). The training-stimulation group was stimulated for 5 consecutive days during training (after daily the last trial, 2 h/day). The probe-stimulation group was stimulated for 2 h just before probe test.

### Morris water maze

Two weeks after surgery, rats performed the Morris water maze test as previously described [11]. The water maze consisted of a circular pool (2 m in diameter) filled with dark water (0.5 m in depth, 25 °C) and a circular black escape platform (0.15 m in diameter) submerged 2 cm below the water surface. The maze tank was located in a dimly lit room with triangular, circular, and square-shaped spatial cues in three quadrants. Rats were placed in the behavioral room for habituation 30 min before testing. All the rats were trained for 5 consecutive days (4 trials/day) with the platform in a fixed position. For each training trial, the rat was given 60 s to reach the platform. Any rat that did not reach the platform within 60 s was led to the platform by the experimenter and allowed to remain on the platform for 10 s. After 48 h from the final training trial, the rats were given a 60 s probe trial without the platform in the pool. Swim paths were recorded using a video tracking system.

### Acetylcholinesterase (AChE) assay

Immediately after the behavioral test, 5 out of 8 rats from the normal group, 4 out of 8 rats from the lesion group, 5 out of 9 rats from the pre-stimulation group, 4 out of 9 rats from the training group, and 3 out of 7 rats from the probe group were anesthetized and the brains were quickly removed to acquire proteins. The frontal cortex (FC, including the cingulate cortex and prelimbic cortex), MS, diagonal band (DB) and hippocampus were dissected with fine forceps from 1 mm thick coronal brain slices. The tissues were homogenized in lysis buffer (Intron, Seongnam, Korea) on ice for 30 min and then centrifuged for 20 min at 12,000 rpm. The protein in the supernatant was measured using the bicinchoninic acid protein assay reagent kit (Pierce, Rockford, IL, USA). The protein samples were stored at −70 °C until analysis. The activity of AChE was determined using the method of Ellman et al. [12] with some modifications as previously described. In brief, 20 µl triplicate samples were mixed with the reaction mixture of 0.2 mM dithiobisnitrobenzoic acid (Sigma, Louis, MO, USA), 0.56 mM acetylthiocholine iodide (Sigma), 10 µM tetraisopropylpyrophosphoramide (Sigma), and 39 mM phosphate buffer (pH 7.2) at 37 °C for 30 min. The optical density was measured at 405 nm.

### Western blotting

The protein sample was the same as the sample used for the AChE assay. Proteins were separated by 10–15% sodium-dodecyl-sulfate–polyacrylamide gels (SDS-PAGE) and transferred onto polyvinylidene fluoride membranes. Membranes were blocked using blocking buffer (5% non-fat dry milk in phosphate buffered saline containing 0.05% Tween 20, PBST) for an hour at room temperature. The membranes were then incubated with primary antibodies overnight at 4 °C. Then, the corresponding secondary antibodies were applied for 1 h at room temperature. Protein was detected with enhanced chemiluminescence solution (GE Healthcare Life Sciences, Uppsala, Sweden) and LAS 4000 mini (GE Healthcare Life Sciences). The intensity of each band was measured using an analysis system (Multi Gauge version 3.0; Fujifilm, Tokyo, Japan). The list of primary antibodies included brain-derived nerotrophic factor (BDNF, 1:1000; Millipore, Temecula, CA), glutamate decarboxylase 65/67 (GAD, 1:1000; Millipore) and ß-actin (1:5000; Sigma).

### Histology

Three out of 8 rats from the normal group, 4 out of 8 rats from the lesion group, 4 out of 9 rats from the pre-stimulation group, 5 out of 9 rats from the training group, and 4 out of 7 rats from the probe group were anesthetized and perfused with normal saline and cold 4% paraformaldehyde. The brains were stored in 4% paraformaldehyde for 3 days at 4 °C and transferred to 30% sucrose for 3 days. Then the brain sections, which were sliced into 30-µm thickness, were stored in a cryoprotectant solution. (0.1 M phosphate buffer, pH 7.2, 30% sucrose, 1% polyvinylpyrrolidone, and 30% ethylene glycol) at −20 °C. Anatomical landmarks from a stereotaxic atlas were used to localize the MS and hippocampus [13].

Cresyl violet staining was performed to confirm the electrode location. The sections were soaked into Cresyl violet for 2–5 min. Fluorescence immunohistochemistry was performed to detect cholinergic neurons and neurogenesis. Sections were blocked with 10% normal horse serum (Vector Labs, Burlingame, CA, USA) and incubated with primary antibodies at the following dilutions: choline acetyltransferase (ChAT, 1:50; Chemicon, Temecula, CA, USA), Sex-determining region Y-Box2 (Sox2, 1:50; Santa Cruz Biotechnology Inc., Santa Cruz, CA, USA), DCX (1:50; Santa Cruz Biotechnology Inc.). After the primary immunoreaction, sections were incubated with secondary antibodies conjugated with Cy3 (1:400; Jackson ImmuonReserch, West grove, PA, USA) or fluorescein (1:400; Thermo, Rockford, IL, USA). Staining on sections was visualized with LSM 700 confocal microscope (Carl Zeiss, Jena, Germany).

### Statistical analysis

A one-way analysis of variance (ANOVA) was used to analyze data from all trials. To evaluate the extent of spatial memory disruption, one-way ANOVAs were used to compare the groups receiving DBS at different time points for latency to reach the platform (training phase), time spent in the target quadrant, time spent in the platform zone, and the number of platform crossings. Using these comparisons between the groups, we aimed to confirm that spatial memory is impaired by 192 IgG-saporin, while DBS delivered at different times can lead to memory improvements. The number of ChAT immunopositive cells was counted in 8 coronal sections per group, located 0.7–1.2 mm posterior to the bregma (immunohistochemistry). The number of Sox2- and DCX-immunopositive cells was counted in 8 coronal sections per group, located 3.0–3.6 mm posterior to the bregma (immunofluorescence). The number of ChAT-, DCX- and Sox2-immunopositive cells are presented as the mean ± standard error of the mean (SEM). The results of the western blotting were normalized to β-actin for each sample and expressed as a percentage of the control values. One way ANOVA followed by a post hoc least significant difference test was used at each time point for statistical analysis. P-values less than 0.05 were considered statistically significant. All statistical analyses were performed with SPSS version 21 (IBM Corporation, Armonk, New York, USA).

## Results

### Cholinergic denervation and electrode location

**Fig. 2:**
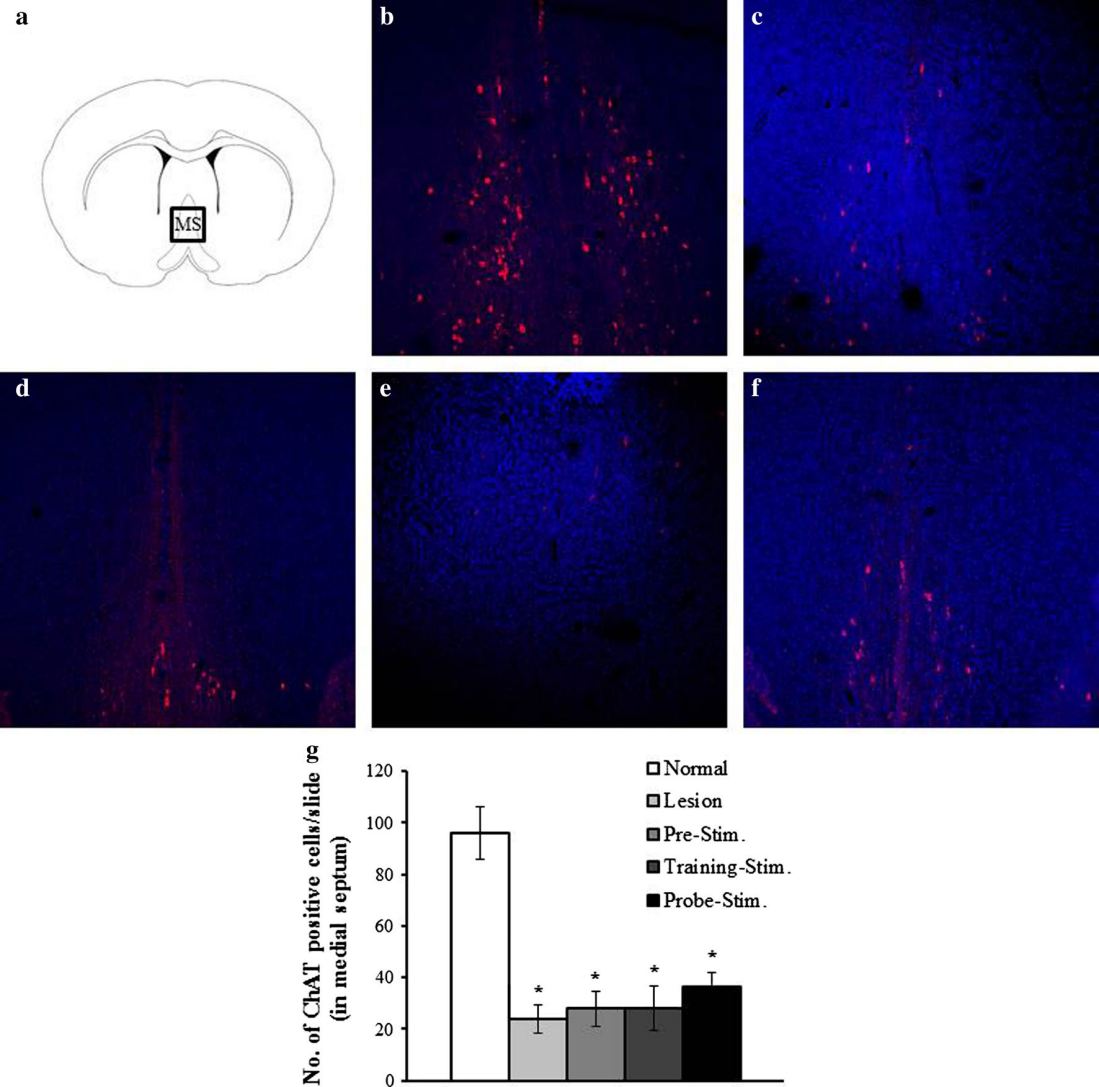
Representative images showing cholinergic lesions after the injection of 192 IgG-saporin. **a** Atlas schematic showing the medial septum. The *square* indicates the location at which the images were taken. **b** The normal group exhibited a large number of choline acetyltransferase (ChAT)-immunopositive neurons (*red*) in the medial septum. The lesion groups (**c**) and all the stimulation groups (**d** pre-stimulation, **e** training-stimulation, **f** probe-stimulation), which were all injected with 192 IgG-saporin, exhibited a loss of ChAT-immunopositive neurons. **g** The number of ChAT-immunopositive neurons was significantly reduced by 192 IgG-saporin (*p* < 0.05)

**Fig. 3:**
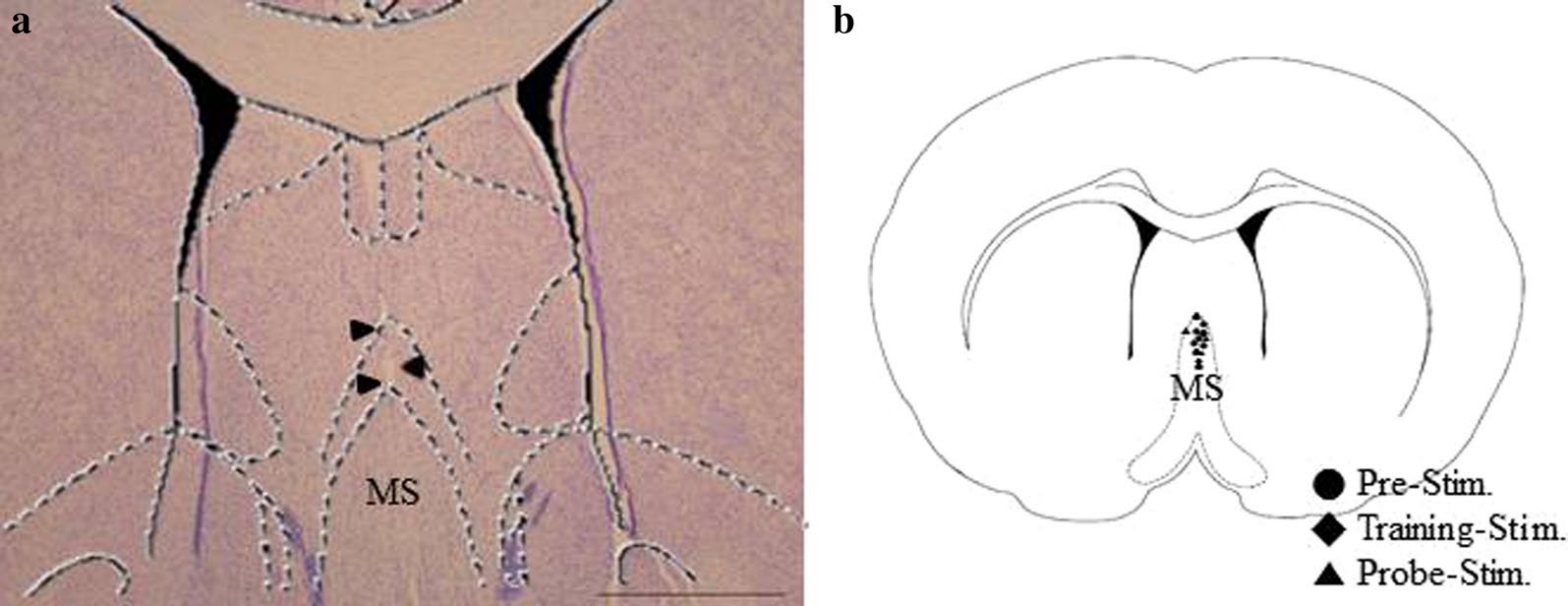
Location of electrodes. A representative stained section and an atlas schematic demonstrating the location of electrodes in the medial septum (MS) are shown. **a** The location of the stimulating electrodes was confirmed using Cresyl violet staining. The *arrowheads* indicate the tract of the electrode. **b** The population of electrode locations on an atlas schematic of MS, where *circle* indicates the location of electrodes in the pre-stimulation group, *diamond* indicates the location of electrodes in the training-stimulation group, and *triangle* indicates the location of electrodes in the probe-stimulation group

Cholinergic denervation was evaluated by counting ChAT immunopositive cells (red) in the MS (Fig. 2). The number of ChAT immunopositive neurons in the normal group was 95.8 ± 10.14. Cholinergic neurons in the normal group were evenly distributed in the MS. In contrast, the number of cholinergic neurons in the groups injected with 192 IgG-saporin was significantly lower (F_4,32_ = 14.6, *p* < 0.0001). The numbers of cholinergic neurons in lesion, pre-stimulation, training-stimulation, and probe-stimulation groups were 24 ± 5.5, 27.75 ± 6.7, and 36.57 ± 5.0 respectively. There wasn’t any noticeable change caused by stimulation. The location of the stimulating electrodes in the MS was confirmed by Cresyl violet staining (Fig. 3).

### Spatial memory is enhanced by stimulation prior to training

**Fig. 4:**
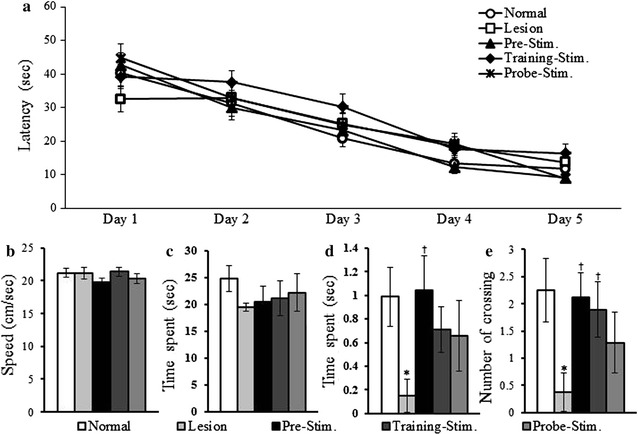
Effects of MS-DBS on spatial memory based on the stimulation time. **a** All the groups gradually acquired the location of the platform. After 48 h from the last training trial, all the groups were administered a probe test. **b** The speed was not different among the groups. **c** The time spent in the target quadrant (in which the platform was placed) was slightly decreased in all the lesion groups. **d** The time spent in the platform zone (in which the platform was placed, 0.15 m in diameter) was significantly decreased in the group with cholinergic lesions compared with normal group (*p = 0.02). However, the time spent in this zone was increased by stimulation. The time spent of pre-stimulation group was significantly increased than lesion group (^†^ *p* < 0.05). **e** The number of platform crossings was also reduced in the cholinergic lesion group and increased in all stimulation groups

The results of the Morris water maze training are shown in Fig. 4a. In all groups, the escape latency decreased from the first day to the last day of training (from over 30 s to less than 17 s). These data demonstrate progressive learning of the hidden platform location. In the Morris water maze probe test, the speed (Fig. 4b) and time spent in the target quadrant (Fig. 4c) were not significantly different between the groups (F_4,36_ = 0.79, *p* > 0.5). However, it is appears that there was spatial memory impairment associated with the cholinergic deficit, as evidenced by the time spent in the target quadrant and the number of platform crossing. The amount of time in the platform zone significantly decreased (F_4,36_ = 1.93, *p* < 0.05) to 15% of the normal group values in the lesion group (*p = 0.028), whereas it only decreased to 72% (*p* = 0.44) and 66% (*p* = 0.38) of the normal group for the training-stimulation and probe-stimulation groups, respectively. The pre-stimulation group spent a similar amount of time as the normal group (1.04 s, *p* = 0.8) in the platform zone. Moreover, the pre-stimulation group significantly spent more time than lesion group (†*p* < 0.05, Fig. 4d). The mean number of platform crossings was 2.25 ± 0.5 in the normal group and 0.37 ± 0.3 in the lesion group (F_4,36_ = 2.09, *p = 0.018). In comparison, the mean number of platform crossing was 2.11 ± 0.4, 1.88 ± 0.5, and 1.28 ± 0.5 for the pre-stimulation, training-stimulation, and probe-stimulation groups, respectively (Fig. 4e). The number of platform crossing was significantly improved in the pre-stimulation and training-stimulation groups compared with that in the lesion group (†*p* < 0.05).

### Cholinergic denervation reduces AChE activity

**Fig. 5:**
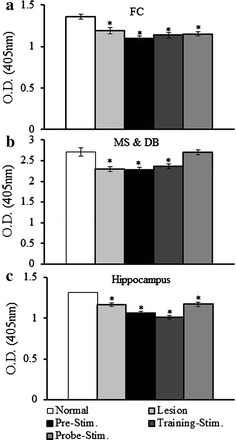
Changes in acetycholinesterase (AChE) activity. **a** AChE activity in the frontal cortex. AChE activity was significantly reduced in the lesion group and all the stimulation groups compared with that in the normal group. **b** AChE activity in the medial septum and diagonal band. AChE activity was restored only in the probe-stimulation group. **c** AChE activity in the hippocampus. Hippocampal AChE activity was significantly reduced in the lesion group and all the stimulation groups compared with the normal group. AChE activity was expressed as the optical density at 405 nm (values represent the mean ± SEM, *p* < 0.05)

There was no restoration of AChE activity associated with MS-DBS, except in the MS and DB of the probe-stimulation group as shown in Fig. 5. AChE activity was significantly reduced in the FC (F_4,10_ = 10.5, *p* < 0.001) of the lesion (*p* = 0.03), pre-stimulation (*p* < 0.0001), training-stimulation (*p* < 0.05), and probe-stimulation groups (*p* < 0.05) compared with that in the normal group. AChE activity also was significantly reduced in the MS and DB (F_4,10_ = 8.9, *p* = 0.002) of the lesion (*p* = 0.002), pre-stimulation (*p* = 0.002), and training-stimulation groups (*p* = 0.007) but was similar to the normal group in the probe-stimulation group. AChE activity in the hippocampus of the lesion (*p* < 0.001) and all stimulation groups (*p* < 0.001) was significantly lower than in the normal group (F_4,10_ = 32.7, *p* < 0.0001).

### Changes in GAD65/67 and BDNF expression

**Fig. 6:**
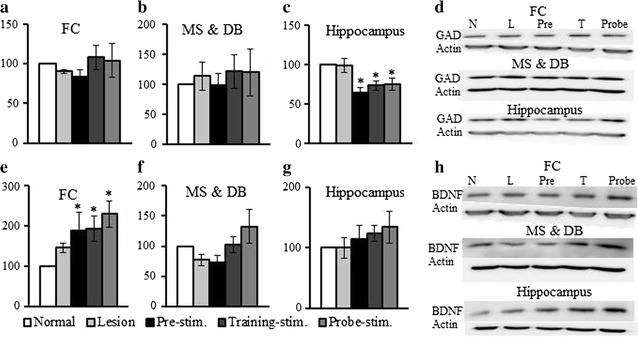
Changes in glutamate decarboxylase (GAD) 65/67 and brain-derived neurotrophic factor (BDNF) expression. The expression level of GAD 65/67 was not significantly different in the frontal cortex (FC) (**a**) or medial septum (MS) and diagonal band (DB) (**b**) for all the groups compared with that in the normal group. **c** The hippocampal level of GAD 65/67 was significantly lower relative to the normal group at all stimulation times. **d** Representative western blotting results. **e** The expression level of BDNF was significantly higher in all the stimulation groups in the FC. BDNF expression also was slightly higher in the MS and DB (**f**) and hippocampus (**g**). **h** Representative western blotting results. The indices are expressed as a percentage of values for the normal group (mean ± SE, *p* < 0.05)

Western blotting was also performed to measure the changes in the expression of GAD65/67 and BDNF as a function of the stimulation time (Fig. 6). The level of GAD65/67 was measured to determine the activity level of GABAergic neurons, which are one of the main components in the projection from the basal forebrain to the hippocampus. The expression level of GAD65/67 was not significantly different in the FC, MS, and DB with the lesion group or stimulation groups compared with the normal group. However, the hippocampal expression level of GAD65/67 was markedly lower (F_4,25_ = 5.86, *p* < 0.05) than that in the normal group in the pre-stimulation (*p* < 0.001), training-stimulation (*p* < 0.05), and probe-stimulation groups (*p* < 0.05). The expression level of BDNF increased in all groups that received stimulation. In the FC, the level of BDNF significantly increased regardless of the stimulation time (F_4,25_ = 2.81, *p* < 0.05). The highest levels of BDNF in the MS, DB, and hippocampus were expressed in the probe-stimulation group. The expression level was higher in the training-stimulation group compared with the pre-stimulation. However, these differences were not significant (*p* > 0.05).

### Neurogenesis is enhanced by stimulation prior to training

**Fig. 7:**
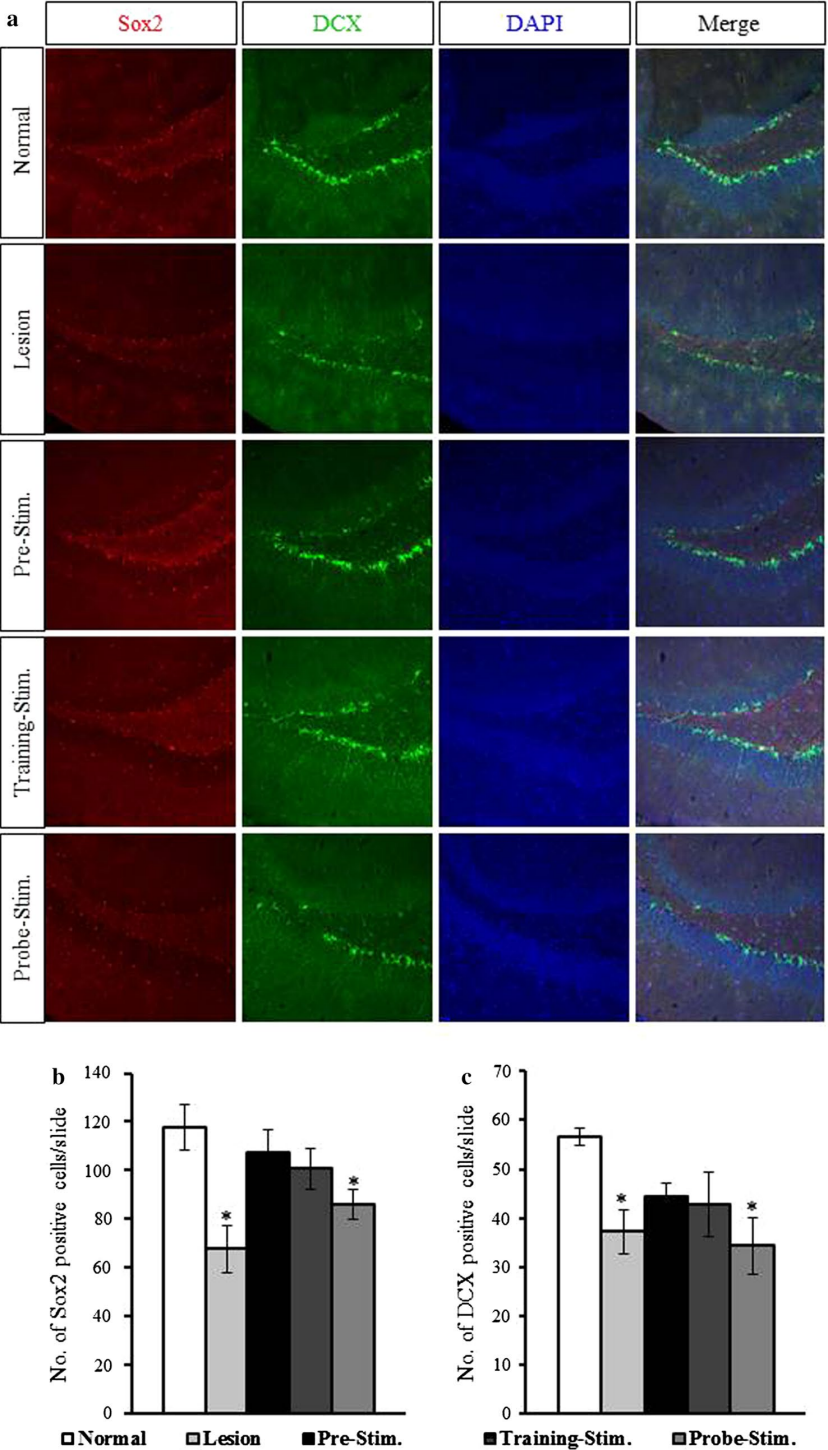
Effects of time-dependent MS-DBS on adult hippocampal neurogenesis. **a** Representative immunofluorescence images reveal the effects of time-dependent MS-DBS on neurogenesis and differentiation. Hippocampal dentate gyrus (DG) sections stained for Sox2 (*red*), DCX (*green*), and DAPI (*blue*) are shown. The number of Sox2- (**b**) and DCX-immunopositive cells (**c**) was significantly lower in the lesion and probe-stimulation groups than in the normal group. However, the numbers of Sox2- and DCX-immunopositive cells were elevated in the pre- and training-stimulation groups (values represent the mean ± SEM, *p* < 0.05)

To evaluate the effect of time dependent MS-DBS on neurogenesis and differentiation, neuronal progenitor cells (Sox2) and neuroblasts or post-mitotic immature neurons (DCX) were quantified (Fig. 7). A significant decrease in the number of Sox2 (F_4,26_ = 5.35, *p* < 0.0001), and DCX (F_4,25_ = 2.09, *p* < 0.05) immunopositive cells was observed in the lesion group compared with that in the normal group (57.3 and 65.7%, respectively). A slight decline in the number of Sox2 and DCX cells was observed in the pre-simulation and training-stimulation groups compared with that in the normal group, but these differences were not significant. The proportion of Sox2- and DCX-immunopositive cells compared with that in the normal group were 90.9 and 78.4%, respectively, for the pre-stimulation group and 85.5 and 75.5%, respectively, for the training-stimulation group. In contrast, the number of Sox2 (F_4,26_ = 5.35, *p* < 0.05), and DCX (F_4,25_ = 2.09, *p* < 0.05), immunopositive cells was significantly lower in the probe-stimulation group compared with that in the normal group (72.9 and 60.5%, respectively).

## Discussion

This study, which was performed to identify the stage of memory at which MS-DBS is the most effective, revealed that MS-DBS prior to training on the Morris water maze test was the most effective in inducing memory enhancement. This memory enhancement may be due mainly to the increase of BDNF expression that was induced by the stimulation. Our study concurs with a clinical study that reported a favorable effect of DBS on disease progression and cognitive function when administered in the early stage of AD [14]. According to the results of the behavioral test, all stimulation time point improved spatial memory, and there were differences in the intensities of these changes. Our understanding of these processes could be improved by further research with various animal models and behavioral tests.

DBS increased BDNF expression mainly in the FC. In addition, increased BDNF expression was maintained for 1 week after the cessation of stimulation. Differences in BDNF expression levels at the stimulation site could be induced by altering the interval between stimulation and sampling. Levels of BDNF in the frontal cortex are correlated with working memory performance [15]. Furthermore, it has been reported that electrical stimulation in this region is associated with BDNF release [16]. BDNF plays a critical role in modulating various neural functions such as membrane excitability, activity-dependent synaptic plasticity, and neurogenesis [17, 18]. Therefore, increasing the level of BDNF using MS-DBS could lead to improved spatial memory. However, it remains unclear what factors determine the level of BDNF expression in different regions.

Hippocampal neurogenesis is thought to be associated with hippocampus-dependent memory [19]. In addition, it has been reported that cholinergic forebrain lesions decrease neurogenesis [20]. The results of this study support the hypothesis that DBS rescues decreased neurogenesis induced by cholinergic lesions. Sox2 is expressed in the adult brain in proliferating precursor cells [21, 22]. DCX is also expressed in late mitotic neuronal precursors and early post-mitotic neurons [23, 24]. The numbers of Sox2- and DCX-immunopositive neurons were reduced by the administration of 192 IgG-saporin. Interestingly, the numbers recovered with pre-stimulation and training-stimulation, which suggest that DBS promotes neurogenesis in the hippocampal dentate gyrus (DG). However, 2 h of MS-DBS was not sufficient to improve neurogenesis, which may be due to the short time interval between stimulation and sacrifice.

Two major neurotransmitter systems of the MS, GABA and acetylcholine regulate hippocampal activity and memory [9, 10]. Moreover, Acetylcholine depresses GABAergic interneurons in the hippocampus [25]. The memory impaired rat model in this experiment was induced by selectively damaging cholinergic neurons in the basal forebrain (including MS and nucleus basalis Meynert), and hippocampus [26]. Therefore, neuronal activity in the hippocampus could be suppressed by the intact GABAergic and damaged cholinergic systems. MS-DBS might regulate the balance between damaged cholinergic and intact GABAergic neurons. As evidenced of decreased GAD expression in the hippocampus, it is also assumed that hippocampal GABAergic suppression by MS-DBS is involved in memory restoration. In addition, GABAergic regulation of neuronal architecture has been reported. A hippocampal GABA_A_ receptor agonist has been shown to impair spatial memory [27]. Prior studies in mutant mice have shown that enhanced GABA_B_ receptor activity reduces the expression of immediate-early genes that encode the protein activity-regulated cytoskeleton-associated protein (Arc) which is essential for synaptic plasticity and memory [28, 29]. Therefore, spatial memory restoration may change synaptic plasticity by suppressing GABAergic activity.

### Limitations of Study

Recently, Lee et al. (2016) have suggested a specific benefit of theta frequency stimulation in traumatic brain injury. Stimulation at 7.7 Hz stimulation in the medial septum improved object exploration and increased hippocampal theta oscillation in adult male Harlan Sprague-Dawley rats. However, 100 Hz gamma stimulation did not enhance performance [30]. Prior to our main study, we had performed a preliminary experiment (data not shown) to investigate the effects of different currents (50 or 100 µA) and different frequencies (10, 60, 130 Hz). In the preliminary experiment, some rats receiving low-frequency stimulation developed convulsions. In contrast, Lee et al. (2016) reported that continuous theta and gamma stimulation did not elicit side effects. This inconsistency may result from differences between the studies in frequency and disease condition. Therefore, as we only used one frequency (60 Hz), this should be considered in the interpretation of our results. In addition, different groups of rats did not receive the same duration of stimulation (2 h/day for 5 days versus only 2 h). Lee et al. (2016) have previously reported that there was no effect of stimulation duration on spatial learning in brain-injured rats. However, it is not clear whether the differences we observed between groups in this study resulted from stimulation timing or stimulation duration Future studies should avoid these limitations by investigating various stimulation frequencies and ensuring that the same stimulation duration is used across groups. In addition, we cannot clearly explain how MS-DBS downregulate hippocampal GABAergic activity. To better understand, more exploring in the other lesion sites and neurotransmitters system is needed.

## Conclusion

MS-DBS (60 Hz, 120 μs, 100 μA) restored spatial memory impairment by increasing the BDNF level, which is associated with neuronal activity and neurogenesis. The pre-stimulation group may have exhibited the most enhancement in memory because it had the longest period of increased BDNF. The enhanced spatial memory associated with DBS might mainly result from increased BDNF level in rather than from direct electrical stimulation of cholinergic or GABAergic neurons. Based on the results of this study, we propose the use of DBS during the early stage of disease to restore spatial memory impairments.

## Abbreviations

DBS: deep brain stimulation
AD: Alzheimer’s disease
MS: medial septum
GABA: gamma-aminobutyric acid
AP: anterior posterior
ML: medial lateral
DV: dorsal ventral
AChE: acetylcholinesterase
FC: frontal cortex
DB: diagonal band
BDNF: brain-derived neurotrophic factor
GAD: glutamate decarboxylase
ChAT: choline acetyltransferase
Sox2: sex-determining region Y-Box2
DCX: doublecortin
ANOVA: a one-way analysis of variance
SEM: standard error of the mean
LTP: long-term potentiation
